# Fungi, feather damage, and risk of predation

**DOI:** 10.1002/ece3.3582

**Published:** 2017-11-08

**Authors:** Zaid Al Rubaiee, Haider Al Murayati, Jan Tøttrup Nielsen, Anders Pape Møller

**Affiliations:** ^1^ Ecologie Systématique Evolution Université Paris‐Sud, CNRS, AgroParisTech, Université Paris‐Saclay Orsay Cedex France; ^2^ Espedal Sindal Denmark

**Keywords:** bacteria, fungi, goshawk, microorganisms, molted feathers, prey

## Abstract

Predation is a powerful selective force with important effects on behavior, morphology, life history, and evolution of prey. Parasites may change body condition, health status, and ability to escape from or defend prey against predators. Once a prey individual has been detected, it can rely on a diversity of means of escape from the pursuit by the predator. Here we tested whether prey of a common raptor differed in terms of fungi from nonprey recorded at the same sites using the goshawk *Accipiter gentilis* and its avian prey as a model system. We found a positive association between the probability of falling prey to the raptor and the presence and the abundance of fungi. Birds with a specific composition of the community of fungi had higher probability of falling prey to a goshawk than individual hosts with fewer fungi. These findings imply that fungi may play a significant role in predator–prey interactions. The probability of having damaged feathers increased with the number of fungal colonies, and in particular the abundance of *Myceliophthora verrucos* and *Schizophyllum* sp. was positively related to the probability of having damaged feathers. In addition, we found a significant correlation between the rate of feather growth of goshawk prey with birds with more fungi being more likely to be depredated. These findings are consistent with the hypothesis that survival and feather quality of birds are related to abundance and diversity of fungi.

## 1 INTRODUCTION

Predation has significant effects on the behavior, morphology, life history, and evolution of prey (Caro, 2005; Curio, 1976; Endler, 1986). Such interactions between predators and prey may result in coevolutionary changes in the phenotype of both interacting parties (Vermeij, 1987).

The factors that determine the risk of predation for individuals, populations, and prey species are poorly known because they require information on observed and expected risk of predation in relation to the variables of interest (Crawley, 1992). Predation risk of hosts may be affected by parasites thereby affecting how predators and prey interact with each other. Such interactions between predators and prey may be direct by changing the phenotype of prey and hence altering the susceptibility of prey to predation. Alternatively, such interactions between predators and prey may be indirect by affecting the phenotypes of prey and hence the risk of predation (Møller, 2008). Indeed, Møller, Peralta‐Sánchez, Nielsen, López‐Hernández, and Soler (2012) showed that the abundance of bacteria living on the plumage of four species of avian prey significantly increased the risk of predation by the goshawk *Accipiter gentilis*. In contrast, Møller et al. (2012) did not show a significant effect of fungi on the risk of predation despite fungi being common microorganisms living on the plumage of birds and pelage of mammals (Hubálek, 2004). We are unaware of any subsequent studies testing for such effects.

Microorganisms can significantly reduce the quality of feathers through feather degradation (Jacob, Colmas, Parthuisot, & Heeb, 2014; Leclaire, Pierret, Chatelain, & Gasparini, 2014; Ruiz‐Rodríguez et al., 2009; Shawkey, Pillai, & Hill, 2009) and hence the flight ability of prey. Any such damage to the plumage would be selected against, with damage to the plumage reaching such extremes as complete degradation and hence disappearance of barbules, barbs, or even loss of entire segments of feathers (e.g., Kim, Lim, & Suh, 2001; Møller et al., 2013; Onifade, Al‐Sane, Al‐Musallam, & Al‐Zarban, 1998; Ruiz‐Rodríguez et al., 2009).

Two categories of microorganisms occur commonly in the plumage of birds. First, birds frequently carry keratinophiles on intact feathers, and some of these keratinophilic fungi are well‐known pathogenic dermatophytes, causing superficial cutaneous infections (dermatophytoses) of keratinized tissues (skin, feathers, hair, and nails) of humans and animals (Deshmukh, 2004), but also direct damage to the plumage of birds. Second, feather‐degrading microorganisms naturally occur in soil. Therefore, prey species that commonly forage on the ground such as gallinaceous birds and thrushes suffer particularly from feather degradation by microorganisms (Burtt & Ichida, 1999). Burtt and Ichida (1999) documented that while ground foraging bird species have a prevalence of 10.7% infested with feather‐degrading bacteria, the prevalence was only 4.7% in foliage‐gleaning species and a mere 2.4% in aerial foragers. This provides evidence for infestation being linked to foraging habitat.

Microorganisms can have strong negative effects on health and fitness of their hosts. Bacteria and fungi are a common cause of disease or mortality in humans and domestic and wild animals (Beaver & Jung, 1985; Benskin, Wilson, Jones, & Hartley, 2009; Evans & Brachman, 1998; Hubálek, 2004; Madigan, Clark, Stahl, & Martinko, 2010; Strauss & Strauss, 2002), and many defense mechanisms have evolved to cope with such infections.

Loss and replacement of old feathers with new ones occurs during molt (Ginn & Melville, 1983). Most birds molt their plumage annually and such feather replacement occurs during a period that may reach 8 months in the wood pigeon (Murton, 1965). Because feathers are important for protection, locomotion, and thermoregulation (Ginn & Melville, 1983), rapid replacement of feathers should reduce the duration of the period when birds experience reduced flight ability caused by growing feathers. However, rapid growth of feathers during molt occurs at the cost of reduced feather quality with speed of molt being traded against the quality of new feathers (Møller & Nielsen, 2017; Pap, Vágási, Czirják, & Barta, 2008). Such a trade‐off may partly be determined by microorganisms because an increased rate of feather growth may be traded against antimicrobial defense of the growing plumage. Thus, we should expect daily growth increments of feathers to be negatively related to the abundance of microorganisms. This implies two possible mechanisms of fungi damaging feathers: (1) during molt by reducing the amount of resources allocated to feathers; or (2) during and after molt by directly damaging feathers by growing or feeding on them. For example, the presence of fungi on the surface of the plumage may directly cause turbulence during flight.

The objectives of this study were to test (1) whether birds with high loads of microscopic fungi are more likely to fall prey to predators than those with few. This question is based on the assumption that more fungi and/or a higher diversity of fungi constitute a greater cost to their hosts. We tested this prediction by investigating the relationship between risk of predation and the abundance of fungi on feathers from wood pigeon, jay *Garrulus glandarius* and blackbird *Turdus merula* that are preferred prey species of the goshawk in our study site in Denmark. The duration of molt reaches 240 days in the woodpigeon, but only 92 days in the jay, 50 days in the song thrush, and 78 days in the blackbird (Ginn & Melville, 1983). All species (including the goshawk) molt during April–August (Ginn & Melville, 1983). In addition, we tested (2) whether the size of daily feather growth increments was related to diversity and abundance of fungi in the plumage. This hypothesis was based on the assumption that defense against microorganisms is traded against rapid feather growth. Finally, (3) we tested whether birds with feathers that developed faster had more damage to their plumage than birds that had slowly developing feathers. While fungi are common microorganisms, there are no studies investigating the relationship between diversity and abundance of fungi and fitness components of prey.

The goshawk is a territorial predator (Cramp & Simmons, 1979; Kenward, 2006), with a distinct division of sex roles during breeding. The smaller male supplies food to the larger female and their chicks, while females incubate the eggs and defend the nests and chicks. Females are about twice as large as males. Prey are caught and killed with claws and usually brought to a traditional site near the nest, plucked, and eaten on the ground (Kenward, 2006).

## 2 MATERIALS AND METHODS

### 2.1 Study sites and field work

We collected flight feathers (primaries, secondaries, and tertiaries) of woodpigeon, jay, and blackbird from exactly the same sites and at the same period of the year. We restricted the feather samples to flight feathers because they can readily be located without long time being allocated to search for feathers. Two categories of feathers were collected from the same forests by the same person (Jan Tøttrup Nielsen), hence avoiding bias in sampling (see also Møller et al., 2012): feathers from plucking sites near 50 nests of goshawks in Northern Vendsyssel (57°10′–57°40′N, 9°50′–10°50′E), Denmark, during April–August 2009 with a mean date of May 16 (*SE* = 2). Male goshawks use traditional eating sites near nests where they bring their prey before presenting it for the offspring or the incubating, brooding, or attending female. Furthermore, molted feathers were collected from the same sites from birds that clearly were alive (because they were molting). All feathers collected were only from recent prey not more than a couple of days old as reflected by the soft structure of feathers. Older feathers rapidly become stiff with rain and exposure to weather. We avoided problems of contamination of feathers by nest contents by only including feathers found on the ground, as were the samples of feathers from live molting birds.

If feathers from multiple individuals were sampled at a site, this could have resulted in pseudo‐replication. However, we emphasize that the death of multiple individuals at a single site due to one or more predators will both result in selective mortality. We also emphasize that each of the 50 nest sites only resulted in inclusion of a single prey individual for each species further reducing the risk of pseudo‐replication.

We assumed that the abundance of microorganisms was consistent across the season. Indeed, Peralta‐Sánchez, Møller, Martín‐Platero, and Soler (2010) have shown for the microbiome on birds’ eggs that the composition is consistent across the breeding season.

### 2.2 Fungal isolation

We isolated fungal species and quantified their abundance on prey feathers and molted feathers. Subsequently, we identified these using the PCR technique. Feathers were cultured directly onto Sabouraud dextrose agar with chloramphenicol (SDA) and moistened with 1 ml of sterilized PBS. The cultures were incubated and examined daily from the third day for fungal growth over a period of 4 weeks. The observed developing mycotic growths under stereoscopic binocular microscope were individually and directly transferred onto Sabouraud dextrose agar with chloramphenicol (50 mg/L). The resulting products were further incubated for 2 weeks to obtain pure isolates for identification purposes.

### 2.3 Fungal identification

All fungal strains were grown on Sabouraud's dextrose agar. A small amount of mycelium was suspended in 200 μl 10 mmol/L Tris–HCl, pH 8.0 in an Eppendorf tube (1.5 ml), and stored in a freezer (−20°C) for further processing.

For molecular identification, genomic DNA was isolated from the fungal strains by using the PowerSoil^®^ DNA Isolation Kit (MO BIO). DNA was eluted in a final volume of 100 μl of 10 mmol/L Tris–HCl, pH 8.5.

PCR amplification was performed in 20–30 μl reaction volume containing 25 μl assay buffer containing 1.5 mmol/L MgCl2, dNTP (10 mmol/L) 0.5–1 μl, 0.5–1 μl of each 0.2 mmol/L primer FR1, 2.5 μl forward primer UF1, Go Taq^®^ G2 DNA polymerase (Promega, Madison, WI, USA) (1.25 μl) 0.5–1 μl, and DNA sample 3–5 μl. The DNA genomic was amplified with initial denaturation at 94°C for 5 min followed by 35 cycles of denaturation for 15 s at 94°C, annealing for 30 s at 55°C, and extension for 1.30 min at 72°C, respectively, and the final extension was carried out at 72°C for 7 min.

Slants of nutrient agar and 40% glycerol stocks were prepared from identified pure culture and stored at 4 and −80°C, respectively, for medium‐ and long‐term preservation.

To visualize and determine the presence or absence of PCR products and to quantify the size of amplified DNA fragments, we performed gel electrophoresis in 1% agarose using 0.5 × TAE buffer (Tris‐Acetate‐EDTA) for 25 min at 100 V. The gel was then stained with Gel Red (BIOTIUM) for 30 min. Images were taken under UV lamp using the photo documentation system IP‐010.SD.

PCR products were sent to Beckman Coulter Genomics, Takeley, Essex, UK for DNA sequencing. The sequence results were processed using the web‐based blasting program, basic local alignment search tool (BLAST), at the NCBI site (http://www.ncbi.nlm.nih.gov/BLAST), and the data were compared with the NCBI/Genebank database.

### 2.4 Feather damage

We quantified feather damage according to whether the tips of feathers were rounded or had indents in the barbules. Hence, we scored feathers as undamaged (a score of 0) or damaged (a score of 1 for indents in barbules) (see also Møller & Nielsen, 2017).

Feather damage or breakage is known to be particularly common in feathers with fault bars, and the risk of mortality due to raptors is considerably elevated in the presence of such bars (Møller, Erritzøe, & Nielsen, 2009). However, we decided against inclusion of a new variable reflecting the prevalence of fault bars because only a small fraction of the birds had fault bars in their plumage.

### 2.5 Statistical analyses

We used the statistical software JMP (SAS 2012) to make all statistical analyses. We log_10_‐transformed all fungal counts after addition of a constant of one to normalize the data. We report total abundance of colonies and species richness for fungi.

In a first test, we used predation as a binomial response variable in a General Linear Model with prey species, fungal abundance, pathogenic or nonpathogenic fungi, the interaction between fungal abundance and pathogenic or nonpathogenic fungi, the interaction between prey species and pathogenic or nonpathogenic fungi, feather growth rate and feather damage as predictor variables. In a second test, we used feather damage as a binomial response variable in a GLM with feather growth rate, fungal abundance, pathogenic or nonpathogenic fungus, the interaction between fungal abundance and pathogenic or nonpathogenic fungi, and the interaction between prey species and pathogenic or nonpathogenic fungi as predictor variables. In a third test, we used feather growth rate as a normally distributed response variable and fungal abundance, pathogenic or nonpathogenic fungus, the interaction between fungal abundance and pathogenic or nonpathogenic fungi, and the interaction between prey species and pathogenic or nonpathogenic fungi.

We estimated effect sizes using Cohen's (1988) guidelines for the magnitude of effects being small (Pearson's *r *=* *.10, explaining 1% of the variance), intermediate (*r *=* *.30, explaining 9% of the variance) or large (*r *=* *.50, explaining 25% of the variance).

## 3 RESULTS

### 3.1 Communities of microorganisms

Among the birds there were 47 woodpigeons, 20 jays, and 20 blackbirds. We isolated 27 fungal species according to identification by PCR technique from feathers of goshawk prey and molted feathers of the same species collected near goshawk nests (Fig. S1). The number of fungal colonies ranged from 0 to 9 with a mean of 2.563 (*SE* = 0.204), *N* = 87. The number of fungal species ranged from 0 to 3, mean = 1.195 (*SE* = 0.085), *N* = 87. Species richness of fungal species, means, *SE*, and ranges of abundance is presented in Table S1.

### 3.2 Fungi and risk of falling prey to goshawks

**Figure 1:**
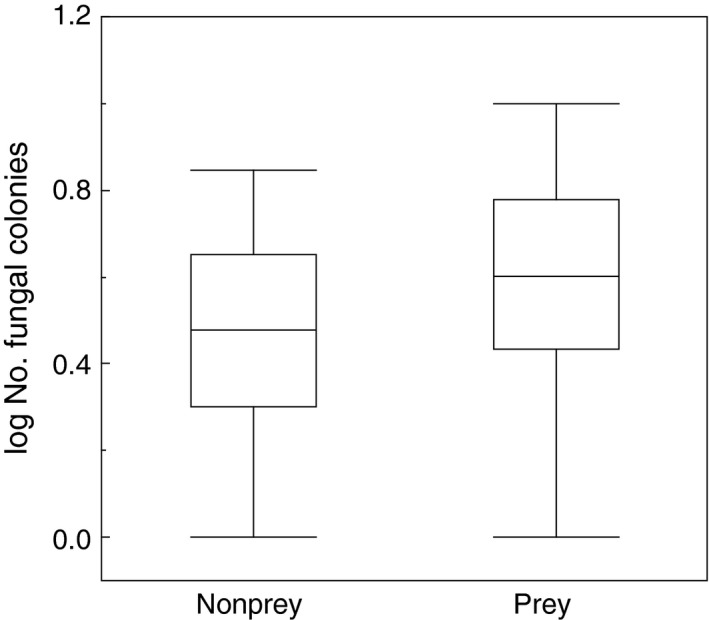
Box plots of mean number of fungal colonies in relation to whether individuals were preyed upon or not. Box plots show means, quartiles, 5‐ and 95‐percentiles, and extreme values

Across all taxa of fungi, there was a significant positive relationship between the likelihood of birds being preyed upon and the mean number of fungi (Figure 1; χ^2^ = 7.65, *df* = 1, *p *=* *.0057, estimate (*SE*) = 2.39 (0.91)). Prey had almost 50% more fungal colonies on their feathers than nonprey that had molted their feathers in the same area and hence were still alive.

**Figure 2:**
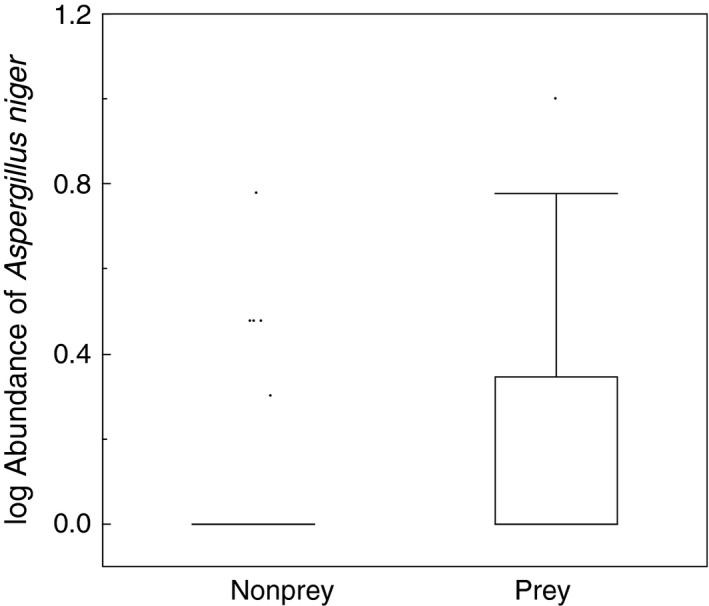
Box plots of abundance of *Aspergillus niger* in relation to whether individuals were preyed upon or not. Box plots show means, quartiles, 5‐ and 95‐percentiles, and extreme values

A GLM with binomial error distribution showed a relationship between the likelihood of birds being preyed upon and the number of colonies of *Aspergillus niger* on feathers (Figure 2; χ^2^ = 4.84, *df* = 1, *p *=* *.028, estimate (*SE*) = 2.18 (1.08)).

### 3.3 Width of daily growth increments, fungi, and species of goshawk prey

**Figure 3:**
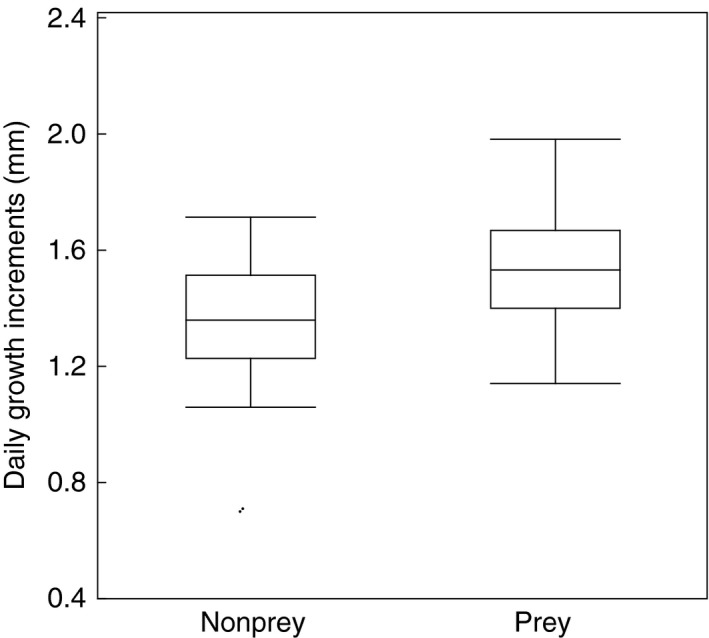
Box plots of daily growth band width of feathers (mm) in relation to whether individuals were preyed upon or not. Box plots show medians, quartiles, 5‐ and 95‐percentiles, and extreme values

The mean width of daily growth increments in all individuals of different species ranged from 0.70 to 1.98 mm with a mean of 1.44 mm (*SE* = 0.25), *N* = 87. Bird feathers with damage had larger daily growth increments than feathers without damage (χ^2^ = 5.91, *df* = 1, *p *=* *.017, estimate (*SE*) = 0.00048 (0.00020)). Birds with wide daily growth increments had a higher probability of falling prey to a goshawk. A GLM with binomial error distribution showed a significant difference in width of daily growth increments between prey and nonprey (Figure 3; χ^2^ = 16.17, *df* = 1, *p *<* *.0001, estimate (*SE*) = 0.005 (0.001)). Therefore, growth increments were wider in prey than in nonprey.

A GLM with normal error distribution showed a significant difference in width of daily growth increments between fungal taxa (*Aspergillus fumigatus, Chaetomium elatum, Chaetomium globosum, Myceliophthora thermophila, Myceliophthora verrucos,* and *Thermomyces lanuginosus;* Table 1; χ^2^ = 30.62, *df* = 6, *p *<* *.0001). We found an overall mean effect size weighted by sample size of 0.38, *SE* = 0.03, 95% confidence intervals 0.31–0.45, Wilcoxon signed rank test = 41718, *p *<* *.0001 (Table 1). This suggests that the mean weighted effect size for the relationship between the width of daily growth increments of feathers and the abundance of different fungal taxa is of an intermediate magnitude (Cohen, 1988).

**Table 1:** Relationship between the width of daily growth increments of feathers (response variable) and the abundance of different fungal taxa (predictor variables). The GLM model with binomial error distribution had the statistics χ^2^ = 30.624, *df* = 6, *p *<* *.0001. Effect size *r* is Pearson's product moment correlation coefficient

Term	Estimate	*SE*	χ^2^	*R*	*z*	*p*
Intercept	2.46	0.01	509.31			<.0001
*Aspergillus fumigatus*	−0.28	0.09	9.70	0.38	0.40	.0018
*Chaetomium elatum*	0.35	0.10	11.56	0.41	0.44	.0007
*Chaetomium globosum*	−0.26	0.07	13.76	0.45	0.48	.0002
*Myceliophthora thermophila*	0.10	0.04	6.52	0.31	0.32	.0106
*Myceliophthora verrucos*	0.18	0.06	5.56	0.29	0.29	.0184
*Thermomyces lanuginosus*	−0.46	0.16	7.94	0.34	0.36	.0048

### 3.4 Damage to feathers and fungi

**Figure 4:**
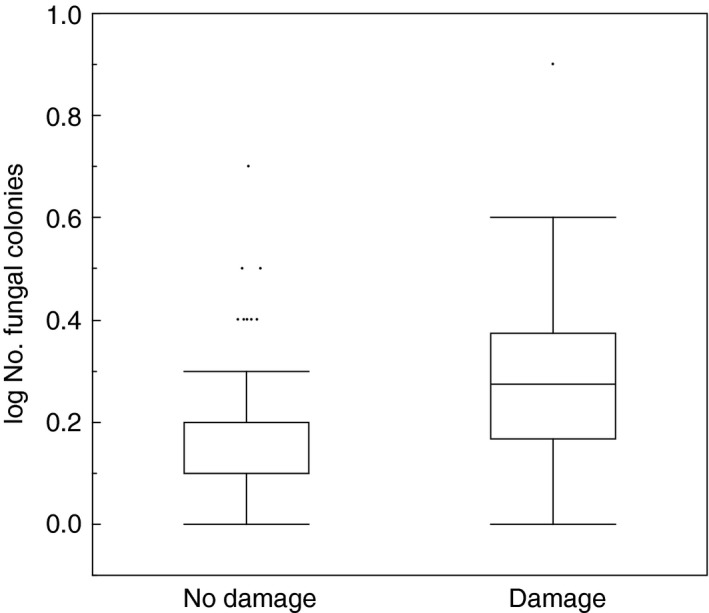
Box plots of total number of fungal colonies in relation to damage of feathers. Box plots show medians, quartiles, 5‐ and 95‐percentiles, and extreme values

The probability of having damaged feathers increased with the number of fungal colonies (Figure 4; χ^2^ = 21.03, *df* = 1, *p *<* *.0001, estimate (*SE*) = 1.41 (0.39)).

## 4 DISCUSSION

The main findings of this study were that the risk for three species of birds falling prey to goshawks was related to the abundance of fungi. Goshawk prey with a specific composition of the community of fungi had higher probability of being preyed upon than goshawks with fewer microorganisms. We found a significant association between the mean number of fungal colonies and whether feathers derived from prey or nonprey. In particular, the abundance of *A. niger* was the best predictor of whether an individual bird was preyed upon. These findings imply that fungi may play a role in predator–prey interactions. In addition, we found a significant difference in mean daily growth increments between prey and nonprey with feathers growing faster in prey. Furthermore, feathers with more fungi differed in size of daily growth increments, and bird feathers with larger growth increments were more likely be from specimens that fell prey to predators. Finally, we found a significant difference in feather damage related to the number of fungal colonies, with a positive relationship between feather damage and the abundance of fungi.

Feathers of wood pigeons, jays, and blackbirds differed in abundance of fungi between prey and live individuals that molted feathers in the same area at the same time. These findings are consistent with a previous study (Møller et al., 2012) showing that goshawks are differentially successful in their capture of prey when prey individuals harbor many bacteria on their plumage. Here, we extended this result to another major group of microorganisms, fungi. This is the first study to show a link between risk of predation and infection of prey with fungi.

Microorganisms of feathers are generally thought to not be harmful (Gunderson, 2008; Shawkey et al., 2009), although they can cause breakage of feather barbs. Hence, the abundance of microorganisms on feathers appears to be a risk factor associated with probability of predation. In wild birds, pathogenic microorganisms are common (Hubálek, 2004; Hubálek & Halouzka, 1996). Thus, predators should differentially capture prey infected by pathogenic microorganisms because that will determine whether an individual survives a predatory pursuit.

There was a large difference in abundance of *A. niger* between prey and nonprey. *A. niger* is a filamentous fungus that is regarded as one of the most important industrial microorganisms that produces many enzymes such as amylases (Mitidieri, Martinelli, Schrank, & Vainstein, 2006), cellulase and xylanase (Couri, da Costa Terzi, Pinto, Freitas, & da Costa, 2000; Farinas et al., 2010), peptidases (Morya, Kumari, & Kim, 2012), and phytases (Bhavsar, Bobbala, Xuan, Föller, & Lang, 2011). For several decades, enzymes from *A. niger* have been used in food production, and there are reports of production of keratinase by *A. niger* strains (Lopes et al., 2008). Hence, we hypothesize that *A. niger* may affect feathers of goshawk prey by reducing feather integrity due to degradation of feather barb keratin. That is also the case for keratinolytic fungi such as *A. niger*, which may reduce fitness of their bird hosts by reduced thermoregulation and flight maneuverability making them more susceptible to predation (Clayton, 1999; Scott & McFarland, 2010; Shawkey, Pillai, Hill, Siefferman, & Roberts, 2007; Swaddle, Witter, Cuthill, Budden, & McCowen, 1996).

Feather quality can affect individual fitness in terms of mate choice, late arrival from migration, delayed timing of reproduction during the breeding season, and escape from predators (Hedenström, 2003; Kose & Møller, 1999; Pap, Tökölyi, & Szep, 2005). The rate of feather growth and feather quality can be affected by many factors such as nutritional status, physiological stress, body condition, and disease (DesRochers et al., 2009; Moreno‐Rueda, 2010; Vágási et al., 2012). Here, we found a positive relationship between the rate of feather growth and the risk of falling prey to a common raptor. These costly effects of rapid molt are condition dependent, so that only birds in prime condition could make a fast molt without compromising their feather quality (Vágási et al., 2012). Here, we found a significant negative relationship between the width of daily growth increments in feathers and abundance of fungal species. In an experimental study of barn swallow *Hirundo rustica* offspring Romano et al. (2011) showed that individuals with parasite infection produced feathers of lower quality. As many fungal species are pathogenetic, as evidenced by our literature review, we suggest that fungal infection in birds during feather growth may cause variation in feather quality.

Feather damage could be used as an indicator of feather quality, as we found a positive relationship between the degree of feather damage and the number of fungal colonies. Among 27 fungal species 14 (52%) secreted keratinase and hence had the ability to degrade feathers (Table S2a). Furthermore, there were 16 (59%) pathogenic fungal species (Table S2b) that can elicit an immune response, and this is costly in terms of energy requirements, but also in terms of reduced feather quality.

The results reported here require experimental manipulation of the abundance of fungi in feathers for formal verification. This could be performed by use of antimicrobial substances on adult feathers, and/or by experimental manipulation of condition for example by experimental manipulation of food availability. Another possibility is to treat adult feathers with antimicrobial agents to determine whether there are negative effects of feather‐degrading fungi.

In conclusion, we have shown that the probability of individual birds falling prey to a raptor increased with the mean number of fungal colonies on feathers. In addition, we found a significant negative relationship between the risk of predation and the abundance of *A. niger*. Moreover, the probability of individuals falling prey to a predator was significantly positively correlated with the width of daily growth increments of feathers from goshawk prey. Finally, we found a positive relationship between damage of feathers and the number of fungal colonies. Hence, we conclude that the abundance of fungi on feathers of goshawk prey, and hence, their microbiome is involved in predator–prey interactions.

## CONFLICT OF INTEREST

None declared.

## AUTHOR CONTRIBUTIONS

APM conceived the idea. JTN collected samples. ZAR, HAM, and APM conducted laboratory analyses. APM and ZAR conducted statistical analyses. All authors wrote the manuscript.
