# Supplementary material for: Fungi, feather damage, and risk of predation
Source: Ecol Evol. 2017 Nov 8;7(24):10797–803. doi: 10.1002/ece3.3582 (PMC5743683; doi:10.1002/ece3.3582)
Supplement: Supplementary file 1 [file ECE3-7-10797-s001.docx]

**Supplementary Materials (SM)**

UU**Table S**U**1**U**.** Identity, range in abundance, mean abundance and standard deviation in abundance of fungi from feathers samples from prey.

| **Fungal** **species** | | | |
| --- | --- | --- | --- |
| Fungal species | Range | Mean | SD |
| *Alternaria sp.* | 0-3 | 1.40 | 0.89 |
| *Antrodia sinuosa* | 2 | 2.00 | 0.00 |
| *Ascomycota sp.* | 0-5 | 3.00 | 2.83 |
| *Aspergillus fumigatus* | 4 | 4.00 | 0.00 |
| *Aspergillus niger* | 0-9 | 2.94 | 2.05 |
| *Aspergillus ustus* | 0-1 | 1.00 | 0.00 |
| *Byssochlamys nivea* | 0-3 | 1.33 | 0.82 |
| *Chaetomium elatum* | 0-2 | 1.17 | 0.41 |
| *Chaetomium globosum* | 0-3 | 1.75 | 0.96 |
| *Chaetomium sp.* | 0-2 | 1.67 | 0.58 |
| *Coniochaeta ligniaria* | 2 | 2.00 | 0.00 |
| *Coniochaeta velutina* | 0-2 | 1.67 | 0.58 |
| *Coprinopsis atramentaria* | 0-4 | 2.33 | 1.03 |
| *Coriolopsis gallica* | 0-4 | 1.78 | 1.20 |
| *Hyphodermella rosae* | 3 | 3.00 | 0.00 |
| *Madurella mycetomatis* | 0-1 | 1.00 | 0.00 |
| *Monascus fuliginosus* | 0-2 | 1.33 | 0.52 |
| *Myceliophthora thermophile* | 0-6 | 2.44 | 1.74 |
| *Myceliophthora verrucos* | 4 | 4.00 | 0.00 |
| *Penicillium sp.* | 2-7 | 3.67 | 2.89 |
| *Pleosporaceae sp* | 0-6 | 2.40 | 2.19 |
| *Preussia sp.* | 0-1 | 1.00 | 0.00 |
| *Psathyrella candolleana* | 2 | 2.00 | 0.00 |
| *Rhizopus oryzae* | 0-4 | 2.00 | 1.41 |
| *Schizophyllum sp.* | 3-4 | 3.50 | 0.71 |
| *Stachybotrys dichroa* | 3 | 3.00 | 0.00 |
| *Thermomyces lanuginosus* | 0-3 | 1.67 | 1.15 |

UU**Table S2a**U**.** Among 27 fungal species, 14 (52%) secreted keratinase.

| ***Fungal taxon*** | ***Reference*** |
| --- | --- |
| *Alternaria sp.* | Saber et al. 2010 |
| *Ascomycota sp.* | Jeyaprakasam et al. 2016 |
| *Aspergillus fumigatus* | Santos et al. 1996 |
| *Aspergillus niger* | Lopes et al. 2011 |
| *Aspergillus ustus* | Anbu et al. 2006 |
| *Chaetomium elatum* | Kaul and Sumbali 1999 |
| *Chaetomium globosum* | Kaul and Sumbali 1999 |
| *Chaetomium sp.* | Kaul and Sumbali 1999 |
| *Madurella mycetomatis* | de Hoog et al. 2013 |
| *Myceliophthora thermophila* | Liang et al. 2011 |
| *Myceliophthora verrucos* | Liang et al. 2012 |
| *Penicillium sp.* | El-Gendy 2010 |
| *Pleosporaceae sp* | Ghosh and Bhatt 2000 |
| *Rhizopus oryzae* | Kumar et al. 2011 |
|  |  |
| **References** |  |
| Saber, W. I. et al. 2010. Keratinase production and biodegradation of some keratinous wastes by *Alternaria tenuissima* and *Aspergillus nidulans*. Research Journal of Microbiology, 5: 21-35. | |
| Jeyaprakasam, N. K. et al. 2016. Determining the Pathogenic Potential of Non-sporulating Molds Isolated from Cutaneous Specimens. Mycopathologia, 181: 397-403. | |
| Santos, R. M. et al. 1996. Keratinolytic activity of *Aspergillus fumigatus* Fresenius. Current Microbiology, 33: 364-370. | |
| Lopes, F. C. et al. 2011. Production of proteolytic enzymes by a keratin-degrading *Aspergillus niger*. Enzyme research, 2011, 1-9. | |
| Anbu, P. et al. 2006. Secretion of keratinolytic enzymes and keratinolysis by *Scopulariopsis brevicaulis and Trichophyton mentagrophytes*: Regression analysis. Canadian Journal of Microbiology, 5: 1060-1069. | |
| Kaul, S., & Sumbali, G. 1999. Production of extracellular keratinases by keratinophilic fungal species inhabiting feathers of living poultry birds (*Gallus domesticus*): A comparison. Mycopathologia, 146: 19-24. | |
| de Hoog, G. S. et al. 2013. Phylogenetic findings suggest possible new habitat and routes of infection of human eumyctoma. PLoS Negl Trop Dis, 7: e2229. | |
| Liang, J. D. et al. 2011. Optimal culture conditions for keratinase production by a novel thermophilic *Myceliophthora thermophila* strain GZUIFR‐H49‐1. Journal of Applied Microbiology, 110: 871-880. | |
| El-Gendy, M. M. A. 2010. Keratinase production by endophytic *Penicillium* spp. Morsy1 under solid-state fermentation using rice straw. Applied Biochemistry and Biotechnology, 162: 780-794. | |
| Ghosh, G. R., & Bhatt, S. 2000. Keratinophilic fungi from Chilka lake-side soil Orissa (India). Indian Journal of Microbiology, 40, 247-254. | |
| Kumar, E. V. et al. 2011. Biodegradation of poultry feathers by a novel bacterial isolate Bacillus altitudinis GVC 11. Indian Journal of Biotechnology, 10: 502-507. | |

UU**Table S2b**U**.**U Among 27 fungal species, 16 (59%) were pathogenic.

| ***Fungal taxon*** | ***Reference*** |
| --- | --- |
| *Alternaria sp.* | Mamgain et al. 2013 |
| *Ascomycota sp.* | [Berbee 2001](http://www.sciencedirect.com/science/article/pii/S0885576501903558) |
| *Aspergillus fumigatus* | Mirhendi et al. 2007 |
| *Aspergillus niger* | Mirhendi et al. 2007 |
| *Aspergillus ustus* | Mirhendi et al. 2007 |
| *Chaetomium elatum* | Hubka 2015 |
| *Chaetomium globosum* | Hubka 2015 |
| *Chaetomium sp.* | Hubka 2015 |
| *Madurella mycetomatis* | Ahmed et al. 2004 |
| *Myceliophthora thermophila* | Farina et al. 1998 |
| *Myceliophthora verrucos* | Revankar and Sutton 2010 |
| *Penicillium sp.* | LoBuglio and Taylor 1995 |
| *Pleosporaceae sp* | Manamgoda et al. 2015 |
| *Rhizopus oryzae* | Bouchara et al. 1996 |
| *Schizophyllum sp.* | Buzina et al. 2001 |
| *Stachybotrys dichroa* | Andersen et al. 2003 |
| **References** |  |
| Mamgain, A. et al. 2013. Alternaria pathogenicity and its strategic controls. Research Journal of Biology 1: 1-9. | |
| Berbee, M. L. 2001. The phylogeny of plant and animal pathogens in the Ascomycota. Physiological and Molecular Plant Pathology, 59: 165-187. | |
| Mirhendi, H. et al. 2007. Identification of pathogenic *Aspergillus* species by a PCR-restriction enzyme method. Journal of Medical Microbiology, 56: 1568-1570. | |
| Hubka, V. 2015. 16 *Chaetomium*. Molecular Biology of Food and Water Borne Mycotoxigenic and Mycotic Fungi, 211. | |
| Ahmed, A. O. et al. 2004. Mycetoma caused by *Madurella mycetomatis*: A neglected infectious burden. The Lancet Infectious Diseases, 4: 566-574. | |
| Farina, C. et al. 1998. Fatal aortic *Myceliophthora thermophila* infection in a patient affected by cystic medial necrosis. Medical Mycology, 36: 113-118. | |
| Revankar, S. G., & Sutton, D. A. 2010. Melanized fungi in human disease. Clinical Microbiology Reviews, 23: 884-928. | |
| LoBuglio, K. F., & Taylor, J. W. 1995. Phylogeny and PCR identification of the human pathogenic fungus *Penicillium marneffei*. Journal of Clinical Microbiology, 33: 85-89. | |
| Manamgoda, D. S. et al. 2015. A taxonomic and phylogenetic re-appraisal of the genus *Curvularia* (Pleosporaceae): Human and plant pathogens. Phytotaxa, 212: 175-198. | |
| Bouchara, J. P. et al. 1996. Attachment of spores of the human pathogenic fungus *Rhizopus oryzae* to extracellular matrix components. European Journal of Cell Biology, 70: 76-83. | |
| Buzina, W. et al. 2001. Development of molecular methods for identification of *Schizophyllum commune* from clinical samples. Journal of Clinical Microbiology, 39: 2391-2396. | |
| Andersen, B. et al. 2003. Molecular and phenotypic descriptions of *Stachybotrys chlorohalonata* sp. nov. and two chemotypes of *Stachybotrys chartarum* found in water-damaged buildings. Mycologia, 95: 1227-1238. | |

**
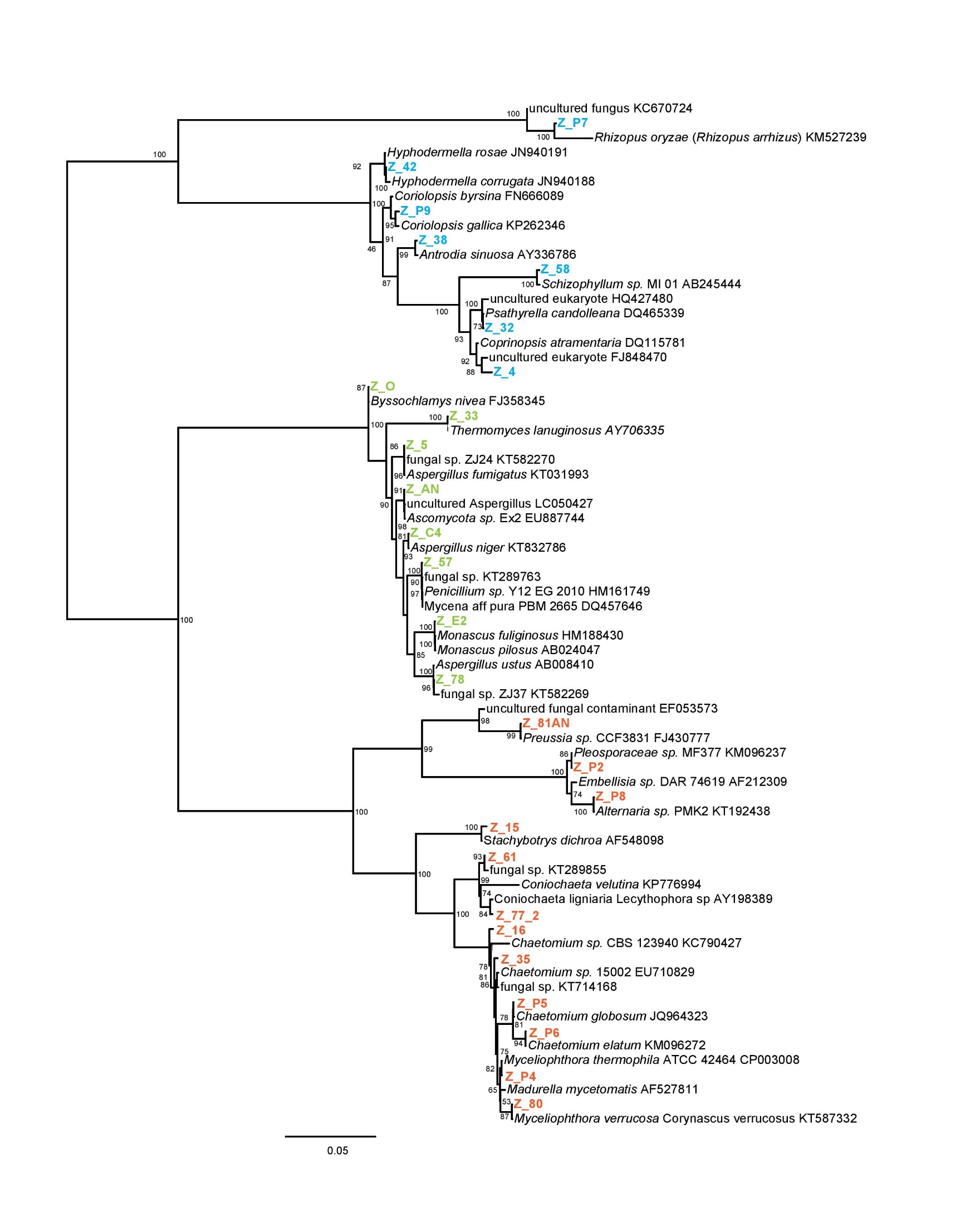
Figure S1.** Phylogenetic tree of 18s rRNA from fungi isolated from feathers of goshawk prey.
